# Filling Roots

**Published:** 1897-07

**Authors:** R. C. Young


ARTICLE IX.

### FILLING ROOTS.

BY DR. R. C. YOUNG.

In Alabama Association.

There are few cases where I believe in immediate root filling. When the pulp is removed by injecting cocain, and this should be done in all single rooted teeth, it is best to fill at once, and here is the only place to use carbolic acid, except in fistulous abscesses. Even if immediate root filling does not give trouble shortly afterward, the tooth breaks down sooner than when the dentine has been thoroughly sterilized. I believe that there is much more under- than over-treatment done. Be careful how you use coagulants. These you can recognize by using a little white of egg in distilled water. Put in your drug, and if it is a coagulant it will cloud the water. Egg albumen and that of the blood are practically the same.

All being ready for filling, put on the rubber-dam making your holes with a red-hot needle. I use an old syringe needle; this makes a hole less liable to tear and hugs the tooth closer, often doing away with the ligature. For root fillings, I like chloro-percha, worked into canal, followed by gutta-percha points. Of course, canal must, in all cases, be as dry as possible. Evans' root dryer is indispensable. Cement is not reliable. It absorbs and becomes offensive. Smell the cement from an old filling, if you want to be convinced. Cotton is worse, lead discolors, and it is only short of a miracle when gold root filling is properly done. Salol is the latest fad, but has not come to stay. If you want an all-day task, try to fill an upper root with it. The crystals are to be packed in canal and then a warm broach inserted, melting salol, which crystallizes and fills root solidly. It melts at one hundred and four degrees. The normal temperature is ninety-eight and a half, fever, often raising it to one hundred and two to four, leaving a very small margin for warm drinks. If a metal filling will conduct heat enough to cause pain, I see no reason why the salol would not melt.

Should the tooth be off color, it should now be restored by bleaching with pyrozone 25 per cent. The dam must be on and root filled. Fill cavity with the pyrozone, evaporate with chip blower. I use pipet with bent point drawn smaller, then they are wrought by heating in alcohol flame till red, then drawing out to size wanted. Or asbestos can be used as a vehicle, but no paper or other fibrous matter should be used, as it lessens the bleaching power. It is unnecessary to seal up in cavity as in using chlorin, etc. I have bleached a badly-discolored tooth in thirty to sixty minutes. And they retain their restored color. One word about opening these tubes. I came near a serious accident with the first I used; it bursted in my hand, throwing contents in my face and pieces of glass all around. Had it gone in my eyes, it would probably have resulted in blindness.

To open,—place tube in vessel of ice-water, then wrap in towel, wet with ice water, file the end or cut with stone, in water, then nip off with pliers and pour into glass stoppered bottle, when all is safe. After bleaching, tooth should be neutralized before filling.—*Items of Interest.*

---

ARTICLE X.

### DENTAL LAWS AND THEIR ENFORCEMENT.

